# Recent Advances in the Synthesis of Peptoid Macrocycles

**DOI:** 10.1002/chem.201705340

**Published:** 2018-02-21

**Authors:** Alexandra M. Webster, Steven L. Cobb

**Affiliations:** ^1^ Department of Chemistry Durham University South Road Durham DH1 3LE UK

**Keywords:** cyclic, macrocycles, peptidomimetics, peptoids, synthesis

## Abstract

Over the past two decades, developing medical applications for peptides has, and continues to be a highly active area of research. At present there are over 60 peptide‐based drugs on the market and more than 140 in various stages of clinical trials. The interest in peptide‐based therapeutics arises from their biocompatibility and their ability to form defined secondary and tertiary structures, resulting in a high selectivity for complex targets. However, there are significant challenges associated with the development of peptide‐based therapeutics, namely peptides are readily metabolised in vivo. Peptoids are an emerging class of peptidomimetic and they offer an alternative to peptides. Peptoids are comprised of N‐substituted glycines where side‐chains are located on the nitrogen atom of the amide backbone rather than the α‐carbon as is the case in peptides. This change in structure confers a high degree of resistance to proteolytic degradation but the absence of any backbone hydrogen bonding means that peptoids exhibit a high degree of conformational flexibility. Cyclisation has been explored as one possible route to rigidify peptoid structures, making them more selective, and, therefore more desirable as potential therapeutics. This review outlines the various strategies that have been developed over the last decade to access new types of macrocyclic peptoids.

## 1 Introduction

### 1.1 Peptide drugs

**Figure 1:**
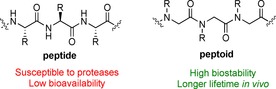
A structural comparison of α‐peptides and α‐peptoids.

Research focused on the development of peptide‐based drugs continues to gather momentum, in part due to the “chemical space” that peptides occupy between small molecules and biologics (e.g. antibodies). In addition properties such as biocompatibility and diversity, both in terms of functionality and structure, make them attractive candidates for a variety of biomedical and therapeutic applications. For example, some peptides (e.g. Nisin A) have been found to be highly active against Gram‐positive and Gram‐negative bacteria^1^ while also appearing to be less susceptible to bacterial resistance than conventional antibiotics.^2^ The versatility in the structure and functionality of peptides enables them to bind specifically to cell receptors, for example, G protein‐coupled receptors (GPCRs), which are responsible for triggering cell signalling responses.^3^ This raises the possibility of using peptides to selectively treat metabolic diseases and different types of cancers,^4^ as well as offering the chance to exploit their targeting properties in areas such as drug delivery^5^ and cellular imaging.^6^ Accordingly, there are over 60 peptide drugs currently approved by the US Food and Drug Administration (FDA), and more than 140 in different stages of clinical trials.^4^ However, despite the promise that peptides offer as therapeutic agents, there are significant obstacles to overcome in terms of their development as commercially viable drugs. In particular peptides may often show a high level of activity in vitro but be completely ineffective in vivo due to rapid degradation by proteases.^7^ A short in vivo half‐life also means that for peptides oral administration is very rarely possible, thus further limiting their utility as drugs. To overcome these barriers, molecules resembling peptides are being developed by many groups in both academia and industry. These molecules are often referred to as peptidomimetics and among these are a class of compounds known as peptoids (Figure 1).^8^

### 1.2 Peptoids

**Scheme 1:**
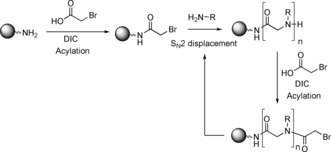
The sub‐monomer method for peptoid synthesis.

Whilst peptoids do share some similarities with peptides, such as biocompatibility and the ability to incorporate different functional groups via their side‐chains, they have significant differences. The side‐chains within a peptoid are moved from the α‐carbon to the amide nitrogen. This structural change means that a peptoid backbone is made up from repeating tertiary amides which imparts an extreme resistance to enzymatic degradation. Furthermore, the shift of the side‐chain means there are no stereogenic centres on the peptoid backbone. The lack of amide backbone protons means that peptoids are more flexible than their peptide counterparts as it is the inter‐ and intra‐chain hydrogen bonding involving the amide proton in peptides that enables the formation of α‐helices, β‐sheets etc. The backbone tertiary amides within peptoids are able to adopt *cis*‐ or *trans*‐conformations and any stable secondary structures are derived purely from steric and/or electronic interactions.^9^ This means that peptoids are not as readily denatured by solvent and temperature changes as their peptide counterparts.^10^ Peptoids are routinely synthesised using the highly flexible sub‐monomer method developed by Zuckermann et al. (Scheme 1). This is a solid phase synthesis approach, which comprises two steps: acylation using a halo‐acetic acid (typically bromoacetic acid), then displacement using a primary amine.9a There are other, less commonly used methods of peptoid synthesis, for example, solid phase monomer synthesis,^11^ and solution phase methods such as ring‐opening polymerisation of *N*‐substituted *N*‐carboxyanhydride monomers^12^ and Ugi 4‐component reactions,^13^ but these are beyond the scope of this review, except for when specific examples have played a key role in accessing macrocyclic peptoids.

### 1.3 Cyclic peptoids

In order to design drugs that interact with a specific target, conformational rigidity is important. As well as increasing the affinity of the compound to the target,^14^ conformational rigidity reduces the likelihood of any off‐target effects that may be due to a lack of specificity.^15^ Limiting off‐target effects is particularly difficult whilst treating certain diseases; the side‐effects that commonly occur during current cancer treatments are well documented.^16^ In nature, one of the ways in which conformational rigidity is achieved is by forming covalent bonds that effectively “staple” the three‐dimensional structure of the peptide in place. For example, nature produces numerous cyclic peptides that exhibit a range of potent biological activities including antibacterial properties.^17^ In many classes of peptides di‐sulphide bridges (between cysteine residues) are commonly used to constrain peptide conformation, and also enhance stability towards degradation.^18^ Taking inspiration from nature, researchers have reported a wide range of synthetic peptides, where cyclisation was used as a strategy to enhance resistance to proteolysis and also effect greater cell penetration.^19^

In linear peptoids the main source of conformational heterogeneity arises due to *cis*‐ *trans*‐isomerisation around the backbone amide bond.^20^ In an effort to access stable peptoid structures there has been increasing interest in new routes to access cyclic peptoids.^21^ As with peptides, cyclic peptoids have been shown in several cases to improve cell penetration and also to enhance antimicrobial activity when compared to their linear precursors.^22^ Cyclisation of linear peptoids restricts the movement of the amide backbone, increasing rigidity and reducing the number of possible conformations. Cyclic peptoids were first synthesised in 2007 by the Kirshenbaum group, and an excellent review by Yoo et al. (published in 2010) summarised the initial work carried out within the field to make peptoid macrocycles.^23^ This current review provides an update on the progress within the field and it focusses on the work carried out from 2010 onwards. The cyclisation strategies have been collated into three general categories: head‐to‐tail, side chain‐to‐side chain and side chain‐to‐tail cyclisation and these are discussed in the context of their possible applications.

## 2 Head‐to‐Tail Cyclisation

**Scheme 2:**
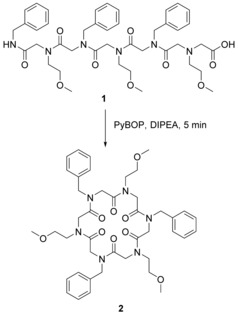
Formation of a peptoid macrocycle (**2**) via head‐to‐tail cyclisation of a linear peptoid hexamer (**1**).

Cyclic peptoids structures have been reported since at least 1969,^24^ however, they were not labelled as such; indeed the term “peptoid” was only coined in the late 1980s^25^ and so the first major report of the synthesis of peptoid macrocycles is considered to be the 2007 paper by the Kirschenbaum group.19a The approach used a head‐to‐tail cyclisation strategy. Ring formation was carried out in the solution phase as a condensation reaction between the N‐terminus and the C‐terminus of the linear peptoid precursor (Scheme 2, where **1** was prepared by cleavage from 2‐chlorotrityl resin).

Linear peptoid chains up to 20 monomers in length underwent rapid room temperature head‐to‐tail cyclisations giving up to 90 % yields after 5 minutes and at moderate dilutions (0.6–3.0 mm). Cyclisation of the peptoid octamer [(*N*phe*N*me)_4_] at concentrations ranging from 0.3 to 78 mm was found to proceed with little accumulation of the unwanted dimeric product formed though intermolecular reaction. Conversely, increased ring strain meant that the tetramer [(*N*phe*N*me)_2_] only cyclised with a 12 % yield after 5 minutes. Notably, Shin et al. managed to crystallise the cyclic hexamer (**2**) and the resulting crystal structure showed that the hydrophobic phenyl side‐chains (*N*phe) oriented on one face of the ring and the hydrophilic methoxy ethyl side‐chains (*N*me) oriented on the other face. This has implications for the future design of high order peptoid oligomers; it may be possible to design more complex peptoids which cyclise to form an ordered, amphiphilic structure.19a

**Scheme 3:**
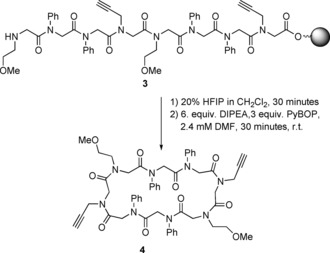
Formation of a cyclic peptoid (**4**) which assembles into a nanotubular structure and is capable of reversibly sequestering water.

**Figure 2:**
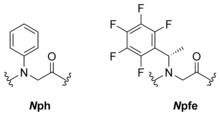
N‐aryl glycine (*N*ph) side‐chain and pentafluorobenzyl (*N*pfe) side‐chain which favour *trans* and *cis* conformations, respectively.

This method of head‐to‐tail cyclisation was used in 2013 by the Kirshenbaum group to make a cyclic peptoid octamer (**4**, Scheme 3) which assembles to form a nanotubular structure capable of reversibly sequestering water.^26^ The linear parent peptoid (**3**) was designed to incorporate side‐chains that would impose a sequence of *cis* (*c*) and *trans* (*t*) amide bond configurations corresponding to *ccttcctt*; a sequence observed in many peptoid macrocycles.19a *N*‐aryl glycine (*N*ph) monomer units have been shown to exhibit a strong preference for a *trans*‐conformation, whilst some *N*‐alkyl (e.g. *N*pfe) monomer units show a preference for *cis* conformation (Figure 2).^27^

**Figure 3:**
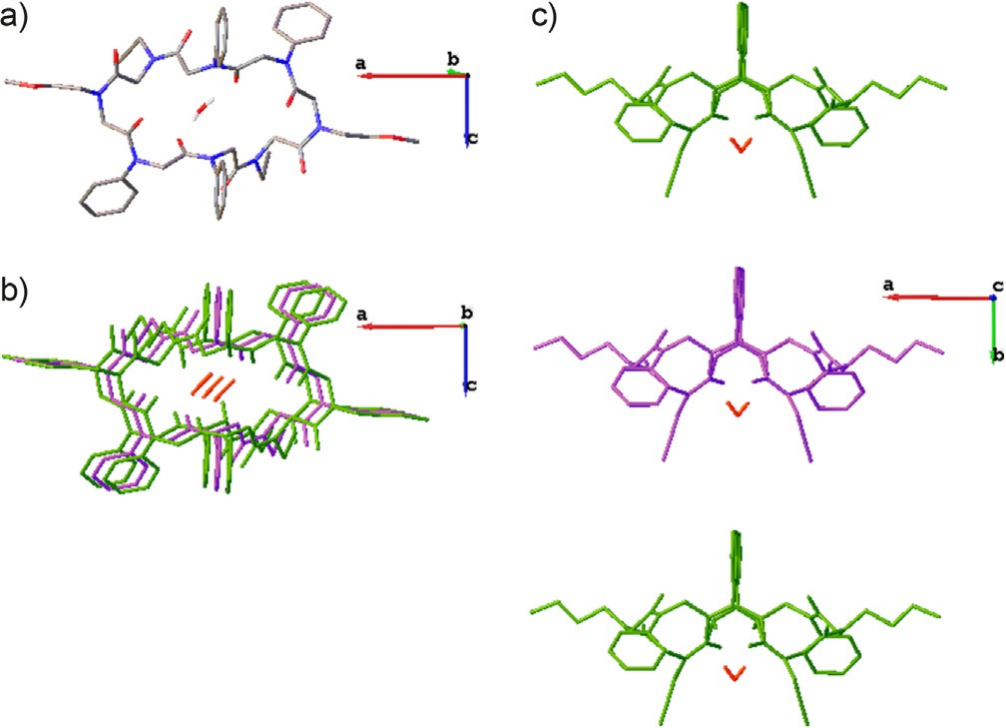
Crystal structure of water‐sequestering peptoid macrocycle: (**4**), a) crystal structure of single molecule with oxygen in red and, nitrogen in blue and hydrogen in white; b) top view of three stacked peptoid macrocycles showing the cavity in which water (red) is sequestered; c) side view of three stacked peptoid macrocycles. Hydrogen is omitted (except for the water molecules) for clarity.

This principle was used to select the monomers in the synthesis of the linear parent peptoid (**3**); the aryl groups (*N*ph) enforced the *trans*‐conformation about the amide bonds whilst the methoxy groups (*N*me) were included to improve water solubility and the propargyl groups (*N*prp) allowed for possible further modification. The crystal structure of the resulting macrocyclic peptoid (**4**) had a conformation that was as predicted, with the alkyl groups allowing a *cis*‐conformation of associated amide bonds and the aryl groups enforcing *trans*‐conformations (Figure 3).

Cyclic peptoid hexamers based on Kirshenbaum's scaffolds have been found to exhibit various interesting properties, including antimicrobial action.^21^, ^22^, ^28^ The De Riccardis group have contributed significantly to this area, highlighting the potential of these cyclic peptoid motifs to take up guest molecules,29a to act as phase‐transfer catalysts,29b to complex metals, including gadolinium,29c and to act as glycosidase inhibitors via formation of iminosugar–cyclopeptoid conjugates.29d,29e De Riccardis and co‐workers have also recently carried out elegant detailed studies on the conformational isomerism that occurs in cyclic peptoids of this type.29f

### 2.1 Small head‐to‐tail macrocyclic α‐peptoids

**Scheme 4:**
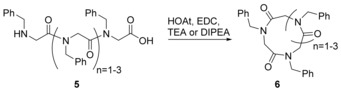
Head‐to‐tail cyclisation of short linear peptoids (**5**) to form small cyclic peptoids (**6**).

Since 2007, efforts have been underway to synthesise smaller (3‐ to 5‐mer) cyclic peptoids, but the yields obtained were often relatively low, particularly for the trimers(<20 %) or conditions were not optimised.^21^, ^30^ Accessing this type of peptoid is desirable given that small cyclic tetra‐peptides have been shown to act as histone deacetylase inhibitors (HDIs).^31^ HDIs have long been used as mood stabilisers and anti‐epileptics, but are now also attracting interest as possible treatments for inflammatory^32^ and parasitic diseases,^33^ as well as cancers.^34^ In 2012, Olsen et al. reported the synthesis of cyclotetrameric peptoid‐peptide hybrids which inhibited class 1 histone deacetylases.^35^ Hoping to provide the tools to eventually make entirely peptoid‐based HDIs, in 2014, Culf et al. optimised conditions for the synthesis of cyclic tri‐, tetra‐ and penta‐peptoids (Scheme 4) and were able to access yields of 80–97 %.^36^

The reactions were carried out in solution using a variety of activators and bases and it was found that a mixture of 1‐Ethyl‐3‐(3‐dimethylaminopropyl)carbodiimide (EDC), 1‐hydroxy‐7‐azabenzotriazole (HOAt) and trimethylamine (TEA) resulted in the best yields. When *n*=1 or 3 the reported yields after overnight, room temperature incubation were 90 % and 97 % respectively (Scheme 4). Under the same conditions, when *n*=2, the reported yield was 38 %, but when EDC and TEA were replaced with 1‐[Bis(dimethylamino)methylene]‐1*H*‐1,2,3‐triazolo[4,5‐b]pyridinium 3‐oxid hexafluorophosphate (HATU) and diisopropyl ethylamine (DIPEA), and the reaction was carried out at 50 °C overnight, the reported yield rose to 80 %. The authors did not elaborate on why the cyclisation of *n*=2 was such a challenge, however they speculated that an increase in temperature improved the yield because of an increased rate of *cis*–*trans* isomerisation about the amide bonds.

**Figure 4:**
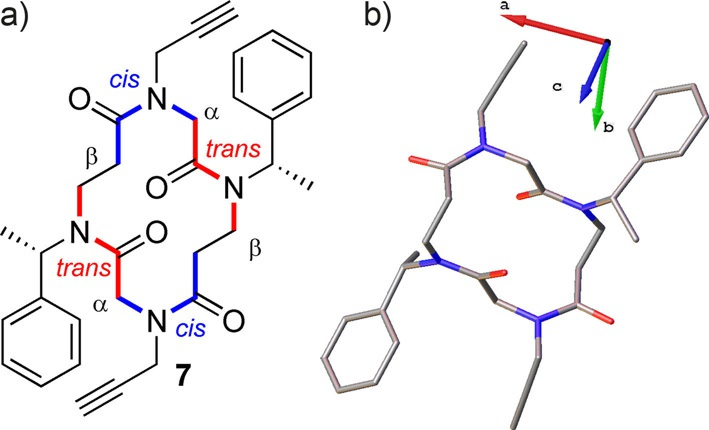
α,β‐cyclic tetrapeptoid (**7**) made by Caumes et al. a) Chemical structure showing alternating *N*spe and propargylglycine monomers; b) crystal structure of **7** showing a *ctct* backbone geometry. Hydrogen is omitted for clarity.

In 2013, Caumes et al. published work investigating the effect of the nature of the side‐chains in the cyclisation of α,β‐tetrapeptoids. They found that the presence of at least one N−Cα‐branched side‐chain was critical for successful cyclisation of these peptoids. Attempts to make cyclic α,β‐tetrapeptoids bearing four propargyl side chains was unsuccessful under almost all conditions attempted, with the most successful attempt resulting in a <10 % yield of the desired cyclic peptoid, and significant amounts (>20 % yield) of the dimeric form. However, when one of these propargyl groups was replaced by an *N*spe monomer, cyclisation occurred. The group was able to obtain a crystal structure of an α,β‐cyclic tetrapeptoid with alternating *N*spe (on the β‐peptoid) and propargyl (on the α‐peptoid) side chains (**7**). The crystal structure showed that the peptoid adopted a β*cis*‐α*trans*‐β*cis*‐α‐*trans* configuration (Figure 4).^37^

### 2.2 Macrocyclic arylopeptoids

**Figure 5:**
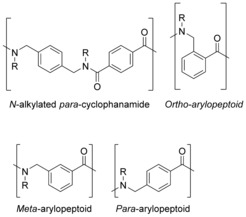
Comparison of the repeating units of *N*‐alkylated *para*‐cyclophanamides and arylopeptoids.

**Scheme 5:**
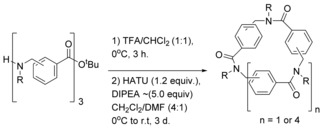
Head‐to‐tail macrocyclisation of arylopeptoids.

An interesting variation of the head‐to‐tail cyclisation approach was reported in 2014 by Hjelmgaard et al. where arylopeptoids were cyclised and found to form higher order nano‐tubular structures.^38^ Arylopeptoids, which are considered to be a subclass of peptoids whereby the backbone is extended by a phenyl ring at each residue, are closely related to *N*‐alkylated *para*‐cyclophanamides (Figure 5). Macrocyclic N‐alkylated *para*‐cyclophanamides, if the R group is a long, hydrophobic chain, form a hydrophobic cavity and thus, these compounds show potential as selective hosts and artificial enzymes. Arylopeptoids can be efficiently synthesised, using the sub‐monomer method, and can readily undergo head‐to‐tail macrocyclisation (Scheme 5) to form rigid, well‐defined structures, similar to N‐alkylated *para*‐cyclophanamides.

The reactions in Scheme 5 were carried out on *ortho*‐, *meta*‐ and *para*‐arylopeptoids. *Para*‐arylopeptoids have a rigid backbone which means that head‐to‐tail cyclisation is challenging. Thus the resulting macrocycles were cyclohexamers (*n*=4, for example, (**8**) rather than cyclotrimers (*n*=1). Formation of the cyclotrimer (**9**) or cyclohexamer (**10**) from the *ortho*‐arylopeptoid is dependent on the nature of the side‐chain; the substituents around the ring are more hindered, so a bulky side‐chain will favour formation of the cyclohexamer. Conversely, the *meta*‐arylopeptoid favours the cyclotrimer (**11**), even with a bulky isopropyl side‐chain.

**Figure 6:**
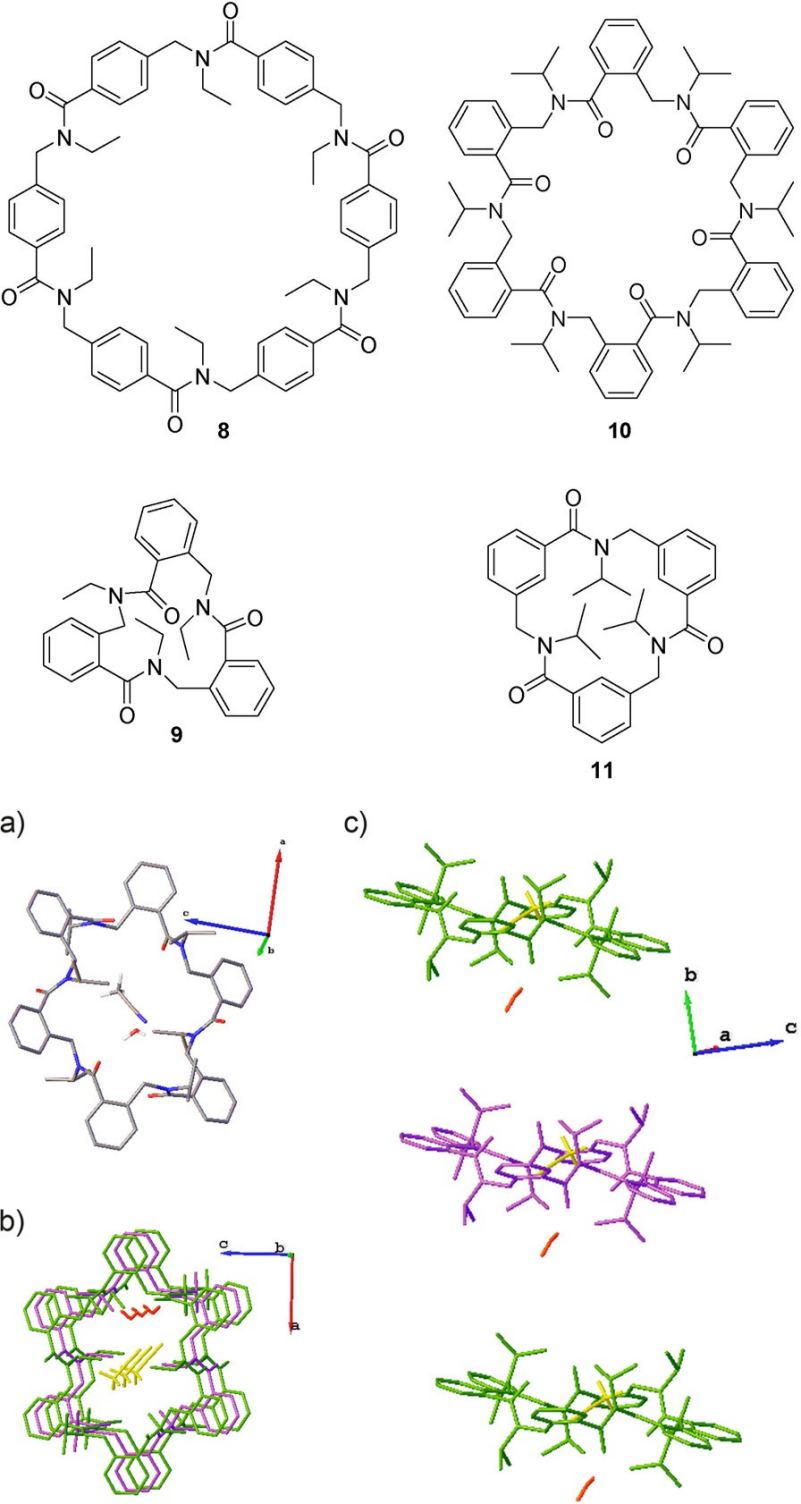
Crystal structure of orthoarylopeptoid cyclohexamer (**10**): a) crystal structure of single molecule with oxygen in red and, nitrogen in blue and hydrogen in white; b) top view of three stacked arylopeptoid macrocycles showing the cavity containing water (red) and acetonitrile (yellow); c) side view of three stacked peptoid macrocycles. Hydrogen is omitted (except for the water and acetonitrile molecules) for clarity.

X‐ray crystallographic analysis of these peptoid macrocycles showed the formation of higher order tubular structures. In the case of the *ortho*‐arylopeptoid, when the side‐chains are isopropyl groups, the cyclohexamer (**10**) which is formed contains one acetonitrile molecule (from the crystallisation solvent) in an interior cavity (Figure 6). The cyclohexamers (**10**) were found to stack to form a tubular array, even in the absence of any hydrogen bonding. It was speculated that a water molecule which bridges two consecutive rings may stabilise the supramolecular assembly. Importantly, the presence of the acetonitrile molecule indicates that the interior cavity of this tubular array is large enough to accommodate a guest molecule, and thus the system has the potential to be developed into a selective host.

### 2.3 Macrocyclic benzylopeptoids

**Scheme 6:**
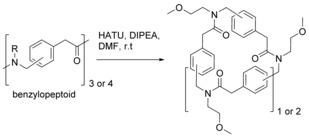
Cyclisation of benzylopeptoids.

Closely related to arylopeptoids are benzylopeptoids (Scheme 6).

Cyclisation was carried out under similar conditions to those used to cyclise the arylopeptoids (Scheme 5 for the arylopeptoids and Scheme 6 for the benzylopeptoids). A solution of the linear benzylopeptoid was added to a solution of HATU and DIPEA in DMF over 6 hours at room temperature and left at room temperature for a further 18 hours. Cyclic *ortho*‐, *meta*‐ and *para*‐benzylo tri‐ and tetra peptoids were successfully synthesised in 26–72 % yields with the *para*‐benzylopeptoid proving most difficult to cyclise for both chain lengths. Subsequent NMR studies showed the ability of all six cyclic benzylopeptoids to complex with Na^+^ ions.^39^

### 2.4 Consecutive Ugi reactions

**Scheme 7:**
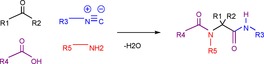
General example of the Ugi 4‐component reaction.

The Ugi 4‐component reaction (U‐4CR) is a multi‐component reaction (MCR) which involves a ketone or aldehyde, an isocyanide and a carboxylic acid (Scheme 7).^40^

The U‐4CR is used to synthesise large libraries of compounds, thanks to the ready availability of a wide range of suitable building blocks. Whilst there are many reports of U‐4CRs being used to make linear peptoids and peptoid hybrids,13a,13d, 41 U‐4CRs have also been used to make and cyclise peptoids.

In 2008, Vercillo et al. reported the syntheses of peptoid macrocycles using consecutive U‐4CRs as a way to generate peptoid‐RGD motifs.^42^ The peptide‐RGD is the tripeptide l‐arginine‐glycine‐l‐aspartate and peptoid‐RGD is the corresponding peptoid sequence (i.e. with the side‐chains moved from the α‐carbons to the backbone amide nitrogen atoms). RGD is common to many peptides involved in cellular recognition^43^ and the RGD loop is recognised by nearly half of all known integrins. Integrins are a family of cell‐adhesion molecules and have key roles in various processes, including thrombosis, metastasis and osteoporosis.^43^ Hence, integrins are attractive therapeutic targets and Vercillo et al. hoped that their peptoid‐RGD‐containing macrocycles could be used in this way.

**Scheme 8:**
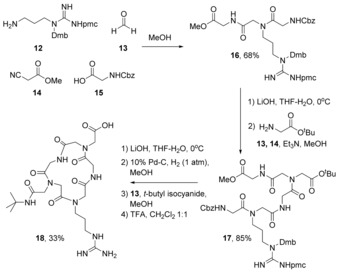
Synthesis of peptoid‐RGD‐containing macrocycle (**18**) by consecutive Ugi reactions.

In order to achieve this, three consecutive Ugi reactions were carried out; the first two, U‐4CRs, yielded the acyclic parent peptoid (**17**) and the third, an Ugi three‐component 4‐centre reaction, gave the macrocyclic peptoid **18** (Scheme 8).^42^

Many RGD peptide macrocycles and non‐peptidic mimics have been shown to be highly active antagonists for a range of integrins.^44^ These RGD peptide macrocycles and non‐peptidic mimics are also selective for particular integrins, due to the conformational rigidity imposed by cyclisation. Unfortunately, studies on the activity and selectivity of **18** were not reported and as such comparison with the macrocyclic peptide analogues is not possible.

## 3 Side‐Chain Cyclisation

### 3.1 Grubbs ring‐closing metathesis

**Figure 7:**
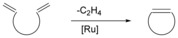
Representative ring‐closing metathesis (RCM).

**Scheme 9:**
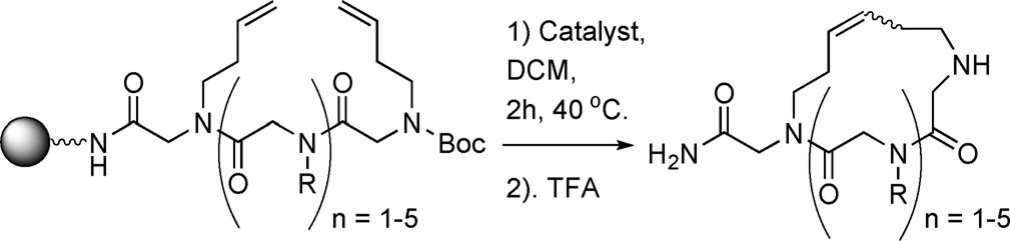
General approach utilised by Khan et al. for the formation of cyclic peptoids using an on resin Ring Closing Metathesis (RCM) strategy.

Olefin metathesis is a widely applied method of carbon‐carbon bond formation using ruthenium alkylidene catalysts. Ring‐closing metathesis (RCM, Figure 7) can be used to form large macrocycles.^45^ RCM has many features that make it attractive for use in the formation of cyclic peptoids; the catalysts are tolerant of a wide variety of functional groups, allowing variation in the side‐chain groups. The catalysts are easily handled, not requiring the use of glove boxes, and the reaction is clean, producing few by‐products, making purification straightforward. In general when transition metal catalysts are used in peptide or peptoid synthesis, solid phase approaches are preferred. This is because carrying out the reaction with the substrate on resin allows a much easier removal of any by‐products including the transition metal. The solid phase synthesis of cyclic peptoids by RCM (Scheme 9) was first reported by Khan et al. in 2011.^46^

Initially, the double bonds in the side‐chain were incorporated through the use of allylamine in the substitution step of sub‐monomer peptoid synthesis. This approach however, only produced the corresponding macrocyclic peptoids in very low yields (10–20 %).^46^ The linear parent peptoid was subsequently altered to extend the length of the alkene‐containing side‐chain by swapping allylamine for 3‐buten‐1‐amine. Various RCM catalysts were also screened, and the most effective one was found to be **19** 
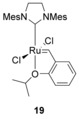
. This combination of longer side‐chains and catalyst **19** produced the target macrocyclic peptoid in 80 % yield. The reaction was carried out both under microwave conditions and at 40 °C on a shaker, with the latter conditions being slightly more efficient, particularly in minimising formation of unwanted dimers.

### 3.2 Thiol‐ene

**Scheme 10:**
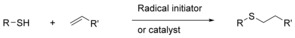
The thiol‐ene reaction.

The thiol‐ene reaction (Scheme 10) is considered a type of “click chemistry” due to its high yields, stereo‐selectivity and fast reaction rates.^47^ There are two mechanisms by which the thiol‐ene reaction may proceed; either by radical addition or Michael addition, catalysed by either a base or a nucleophile.

**Scheme 11:**
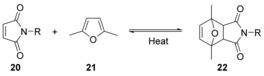
Protection of maleimides by Diels–Alder reaction with 2,5‐dimethylfuran.

The thiol‐ene reaction has been used to cyclise peptides, using a maleimide (**20**) as the source of the double bond.^48^ Non‐protected maleimides can only be incorporated at the end of the chain since they are labile to the nucleophilic bases that are used in peptide/peptoid synthesis. 2,5‐Dimethylfuran (**21**) can be used to protect maleimides (Scheme 11); 2,5‐dimethylfuran (**21**) reacts with the maleimide (**20**) by Diels–Alder cycloaddition. The protected maleimide (**22**) can then be deprotected by simply heating.

**Scheme 12:**
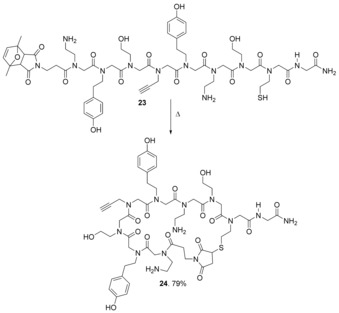
Peptoid cyclisation using the thiol‐ene reaction.

**Scheme 13:**
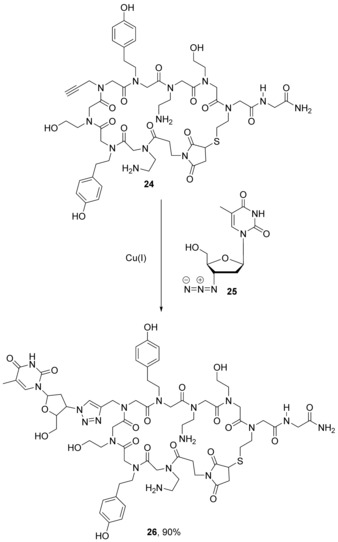
Conjugation of a nucleoside to a cyclic peptoid by Huisgen condensation to form an AZT‐containing cyclic peptoid (**26**).

In principle, protection of the maleimide in this way allows it to be incorporated at any place in a peptide/peptoid chain; however, in the report by Elduque et al., maleimido group inclusion was at the N‐terminus only (**23**). After cleavage from the resin, the maleimido group was deprotected and cyclisation occurred in the same step to give cyclic peptoid **24** (Scheme 12). In the same paper the cyclised peptoid (**24**) was modified with a nucleoside via Huisgen reaction between the alkyne side‐chain and 2′,3′‐dideoxy‐3′‐azidothymidine (AZT, **25**) (Scheme 13).^48^ AZT (**25**) is an *anti*‐retroviral drug used to treat HIV/AIDS.^49^ At high doses, AZT is associated with side effects such as anaemia, neutropenia, hepatotoxicity, cardiomyopathy and myopathy. This limits the dose that can be given to patients, and this means that some HIV replication still occurs. This allows resistance to develop so that, ultimately, the progression of the disease is only slowed.^50^ Development of resistance is slowed by combining AZT with other anti‐retroviral medicines. Conjugation of AZT to cyclic peptoids is of interest to see whether cell uptake and subsequent interaction with components of the cell is improved, or different.

### 3.3 Copper(I)‐catalysed azide‐alkyne cycloaddition (CuAAC)

**Scheme 14:**
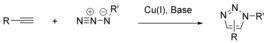
Copper(I)‐catalysed azide‐alkyne cycloaddition (CuAAC).

Copper(I)‐catalysed azide‐alkyne cycloaddition (CuAAC) refers to a 1,3 dipolar cycloaddition between an azide and an alkyne to give a 1,2,3‐triazole (Scheme 14). CuAAC is considered a “click” reaction, and is catalysed by a Cu^I^ compound in the presence of a non‐nucleophilic base.^51^ It is a high yielding and versatile reaction since the required functional groups are easily incorporated into a variety of compounds.

**Scheme 15:**
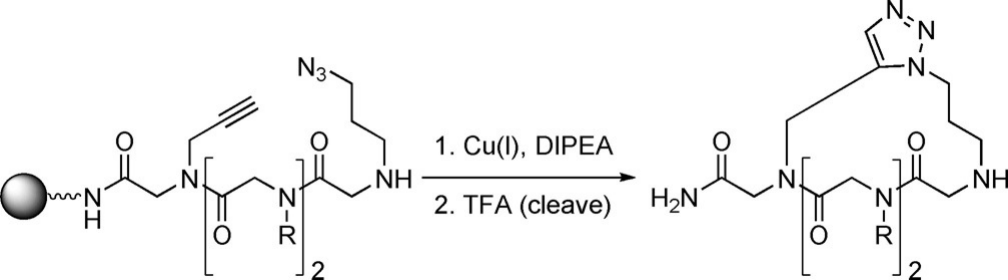
CuAAC to form a cyclic peptoid.

**Scheme 16:**
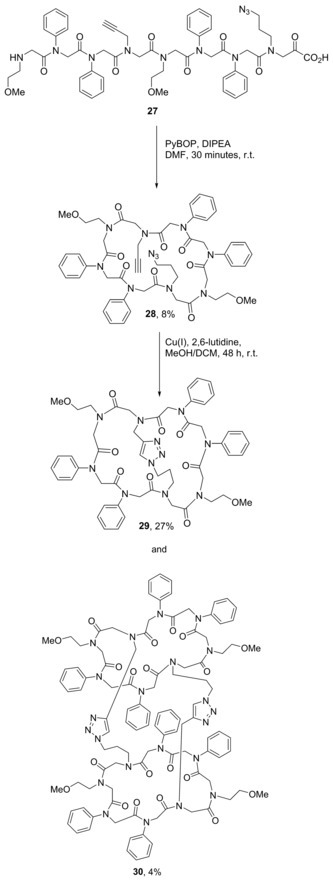
Formation of a bicyclic peptoid (**29**, major product) using CuAAC, and the homodimeric, doubly crosslinked peptoid (**30**, minor product).

CuAAC as a method to cyclise peptoids was first reported in 2007 by the Kirshenbaum group (Scheme 15).^52^ This on‐resin reaction was used as a way to “staple” helical peptoid chains in order to rigidify the structure. This approach was reviewed extensively in the 2010,^23^ and will not be covered in detail here. However, in 2012, the Kirshenbaum group used CuAAC in the solution phase to form a novel bicyclic peptoid scaffold (Scheme 16).^53^

A linear peptoid containing both azide and alkyne groups within the monomer side‐chains (**27**) was first synthesised, cleaved from the resin and then cyclised by head‐to‐tail condensation between the N‐terminus and the carboxylic acid‐terminus to form the monocyclic peptoid (**28**). Bicyclic peptoid **29** was then formed by CuAAC between the side‐chain alkyne and azide groups. This intramolecular reaction was the major reaction pathway under dilute conditions, giving a yield of 27 % but formation of the homodimeric, doubly crosslinked peptoid (**30**) with a yield of 4 % was also observed.

**Figure 8:**
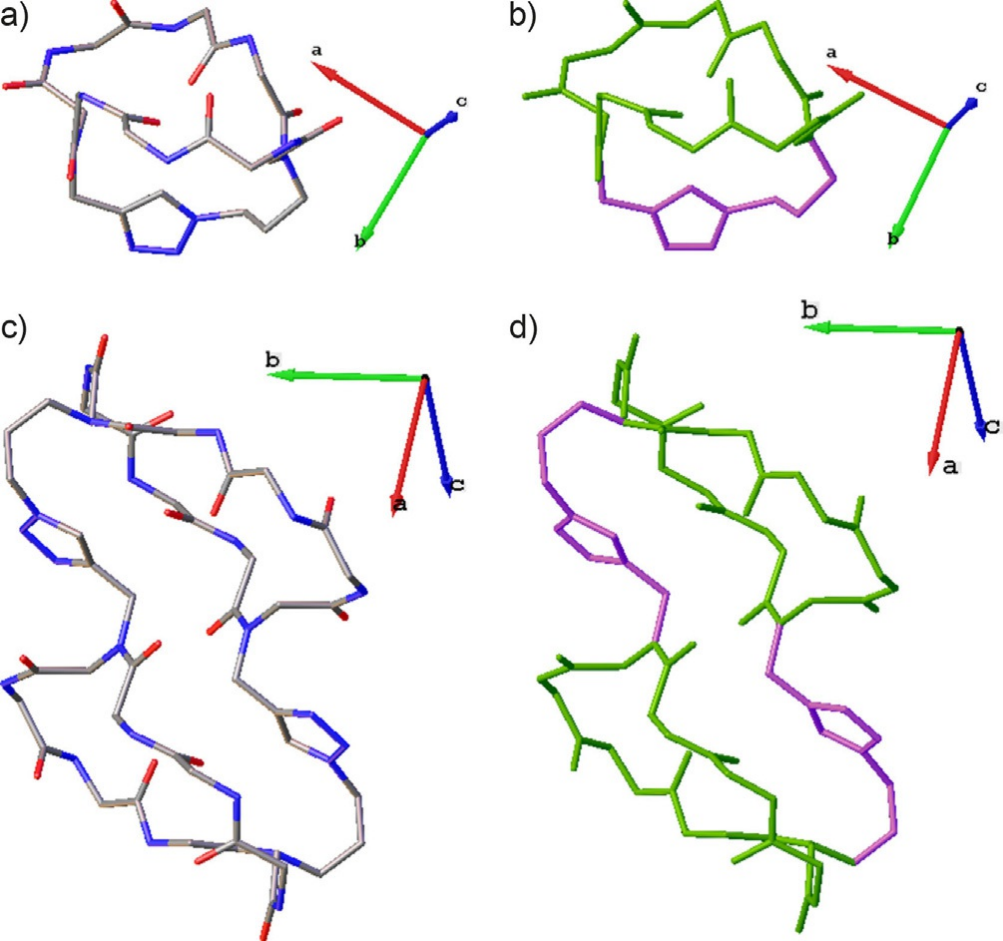
Crystal structures of bicyclic peptoid cyclooctamer (**29**) and the homodimeric, doubly crosslinked peptoid (**30**): a) crystal structure of cyclooctamer (**29**) with oxygen in red and, nitrogen in blue and hydrogen in white; b) crystal structure of (**29**) highlighting the triazole and bridging side‐chains (purple) and the original cyclic peptoid structure obtained via head‐to‐tail macrocyclisation (green); c) crystal structure of the homodimeric, doubly crosslinked peptoid (**30**) with oxygen in red and, nitrogen in blue and hydrogen in white; d) crystal structure of (**30**) highlighting the triazole and bridging side‐chains (purple) and the original cyclic peptoid structures obtained via head‐to‐tail macrocyclisation (green). Non‐bridging side‐chains and all hydrogen atoms are omitted for clarity.

Crystal structures of **29** and **30** were obtained (Figure 8). Whilst **30** appeared to exist in only one configuration, bicyclic peptoid **29** was found to be a mixture of two backbone conformations and further investigation determined the conformation of the monocyclic peptoid to be the main factor contributing to the conformation of the resulting bicyclic peptoid. The formation of bicyclic peptoids through the use of two different cyclisation approaches has not yet been widely exploited but it has the potential to unlock more complex, constrained peptoid conformations.

## 4 Side Chain‐to‐Tail Cyclisation

### 4.1 Triazine‐bridged cyclic peptoid–peptide hybrids

In 2010, Lee et al. synthesised a library of sequencable cyclic peptoid‐peptide hybrids of 3 to 10 residues and later used a similar approach to synthesise an anticancer cyclic peptoid‐peptide hybrid.^54^

**Scheme 17:**
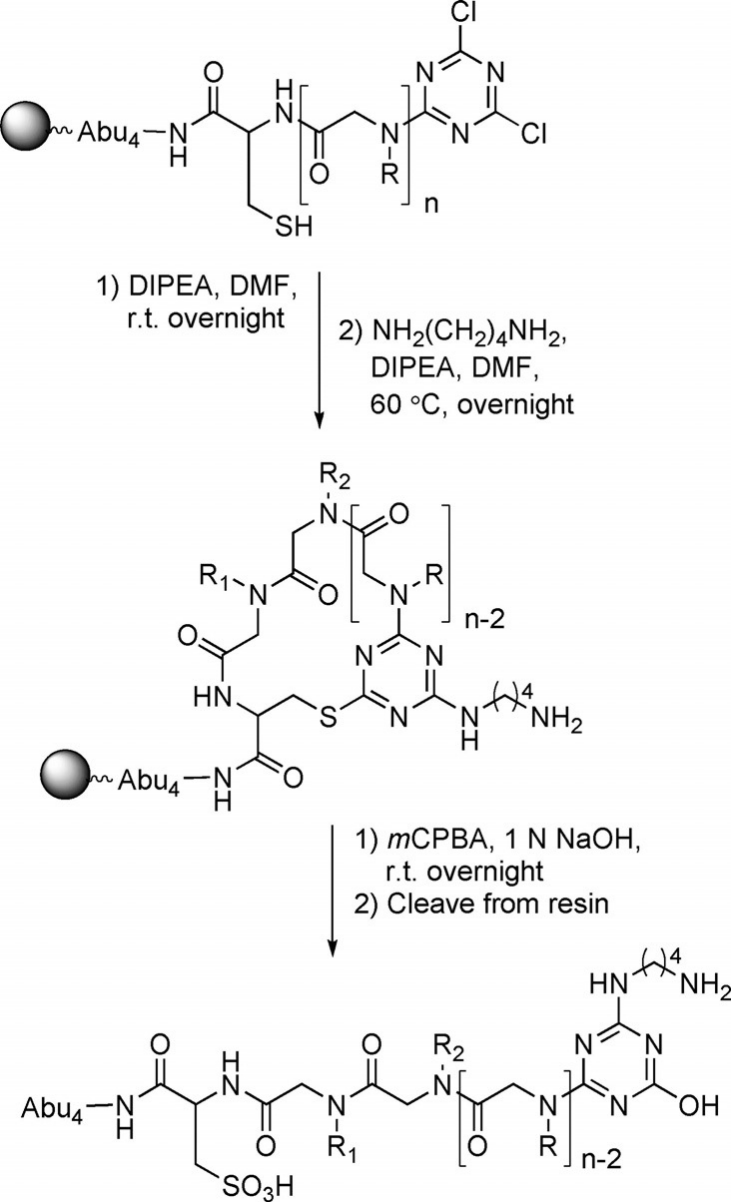
Cyclisation of a cysteine‐containing peptoid–peptide hybrid.

The systems were designed to allow sequencing of hit compounds from high‐throughput screening methods, such as one‐bead‐one‐compound (OBOC). Linear peptoid‐peptide hybrids were made containing a cysteine residue, and capped at the end with cyanuric chloride. Cyclisation was carried out in the presence of DIPEA overnight at room temperature (Scheme 17). The ring could then be opened by incubating the resin‐bound material with *m*CPBA and NaOH overnight at room temperature to yield a linear peptoid‐peptide hybrid that, on cleavage from the resin, could be sequenced by tandem mass spec (MS/MS). The efficiency of the cyclisation reactions was investigated by analytical HPLC and whilst yields were not reported, the purity of the cyclic peptoid–peptide hybrids ranged from 77 to 88 %, generally improving as the sequence got shorter. The authors also reported no detectable amounts of the starting linear peptoid‐peptides, or dimerisation/oligomerisation products.54a In 2016, the same group used this method of cyclisation to synthesise a cyclic compound which inhibited Skp2/p300 interaction, triggering cell apoptosis in cancer cells.54b

**Scheme 18:**
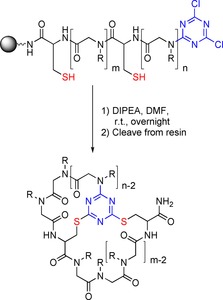
Synthesis of a triazine‐bridged bicyclic peptoid–peptide hybrid.

This method of cyclisation was later expanded to make triazine‐bridged bicyclic peptoid‐peptide hybrids.^55^ In this system, two cysteine residues were incorporated into the sequence, one as the first residue and the second as either the fifth, sixth or seventh residue. Once again, the linear sequences were capped with cyanuric chloride and cyclisation proceeded by incubation of the resin‐bound peptoid–peptide hybrid with DIPEA in DMF overnight at room temperature (Scheme 18). HPLC analysis of the crude reaction products showed efficient conversion of the linear material to the bicyclic peptoid–peptide hybrids with purities of 89–96 % and no detectable by‐products.^55^

### 4.2 Nucleophilic substitution

**Scheme 19:**
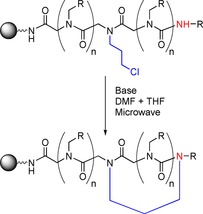
A general overview of the side‐chain‐to‐tail ring formation strategy developed by the Maayan group.

In 2015, Kaniraj and Maayan reported a high yielding side chain‐to‐tail method of preparing cyclic peptoids. The linear parent peptoid includes a chloride side‐chain that reacts with a secondary amine at the terminus of the peptoid chain by substitution under basic conditions (Scheme 19).^56^

The cyclisation reaction was carried out whilst the peptoid was still on resin, meaning that protecting groups on any side‐chain functionalities could be removed at the same time as the peptoid was cleaved from the resin. This allowed for the inclusion of a wide range of functional groups in the peptoid chain. Cyclisation was shown to readily occur when the propyl chloride side‐chain was located in various positions on the peptoid chain giving access to ring sizes as small as 4 and as large as 19.

**Scheme 20:**
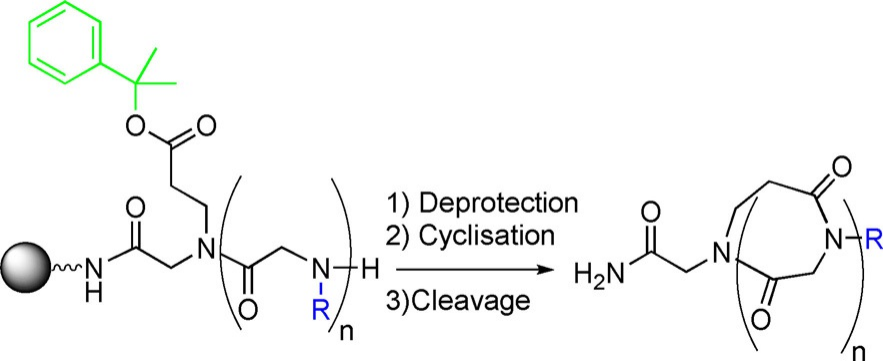
Side chain‐to‐tail macrolactamisation strategy recently reported by Park et al.

As previously discussed the Kirshenbaum group have pioneered the application of a head‐to‐tail macrolactamisation strategy but macrolactamisation can also be used in a side chain‐to‐tail cyclisation (Scheme 20).^57^ However, unlike in the head‐to‐tail approach, the side chain‐to‐tail method allows ring formation to be carried out whilst the peptoid is still on the resin. Using this approach Park et al. were able to prepare macrocyclic peptoids ranging in ring size from 19 atomic members to 55 atomic members. The 55 atom peptoid macrocycle was, at the time of the work by Park et al. the largest peptoid macrocycle reported. Park et al. reported that the efficiency of macrolactamisation varied depending on the ring size and reaction time (6–12 hours). The sequences chosen for the peptoids were based on linear and cyclic peptide sequences known to inhibit the interaction between apolipoprotein E and amyloid‐β; a cause of Alzheimer′s disease, though whether the cyclic peptoids actually interacted with either target was not reported.

**Scheme 21:**
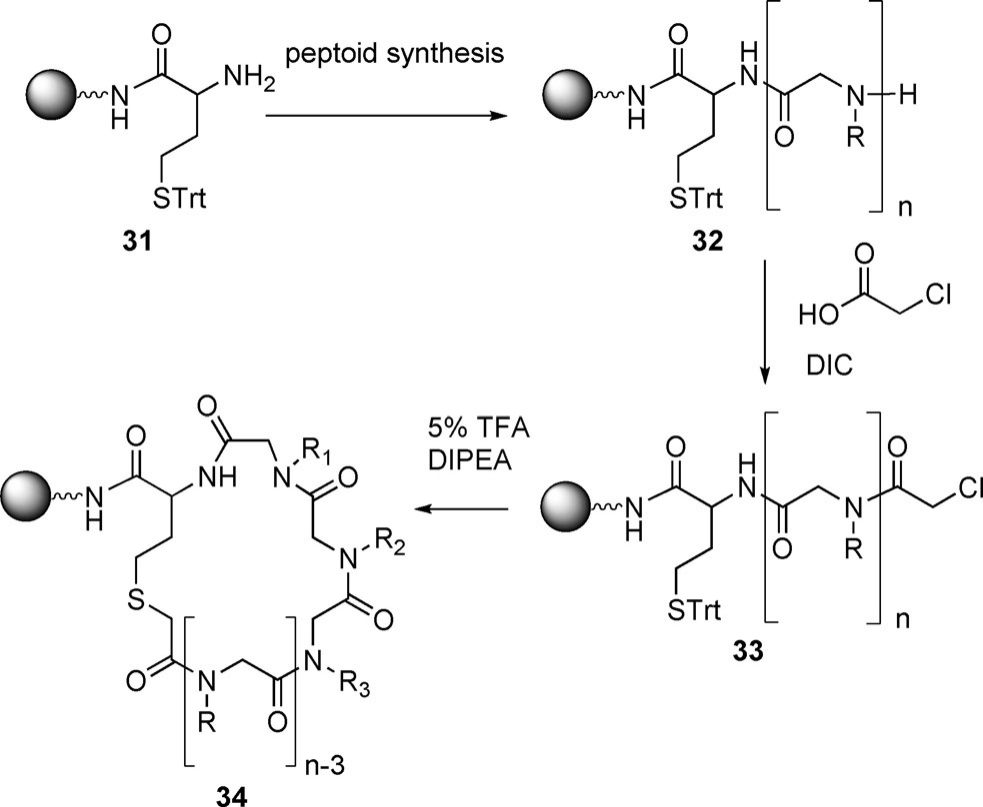
Synthesis of a cyclic peptoid–peptide precursor (**34**).

In terms of characterisation, the sequencing of peptoids can be problematic. In 2014, the successful sequencing of peptoids was achieved by first preparing and cyclising a linear peptoid–peptide hybrid on resin (Scheme 21).

**Scheme 22:**
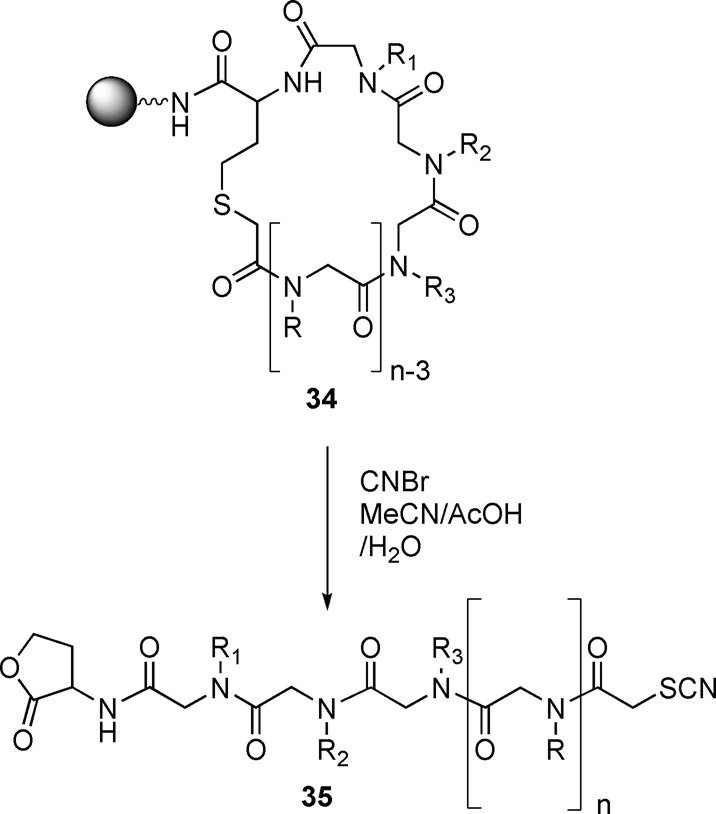
Cleavage of cyclic peptoid–peptide (**34**) to give the tagged linear peptoid (**35**).

A homocysteine was incorporated into the sequence as the first residue, giving **31. 32** was then synthesised by the sub‐monomer method and the N‐terminus chloroacetylated (**33**). Subsequent deprotection of the homocysteine sulfur protecting group and base‐mediated cyclisation gave a cyclic peptoid–peptide hybrid (**34**) which, when cleaved from the resin, generated a linear peptoid that was tagged at each end. The incorporation of two different end groups enabled sequencing by tandem mass spec. The thioether could be oxidised by mCPBA to yield the linear peptoid. However, due the strong oxidising ability of mCPBA, other functional groups in the peptoid were also affected. In order to prevent side reactions with other functional groups, the peptoid is synthesised on Tentagel S NH_2_ resin and CNBr can be used to cleave the peptoid and open the ring (Scheme 22), giving the tagged linear peptoid (**35**).^58^

### 4.3 Suzuki cross‐coupling

**Scheme 23:**
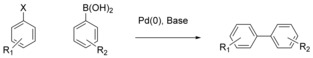
The Suzuki cross‐coupling reaction.

The Suzuki reaction represents a versatile method of synthesising carbon‐carbon bonds. The reaction involves palladium‐catalysed elimination of a boronic acid and halide in the presence of base (Scheme 23).^59^

**Scheme 24:**
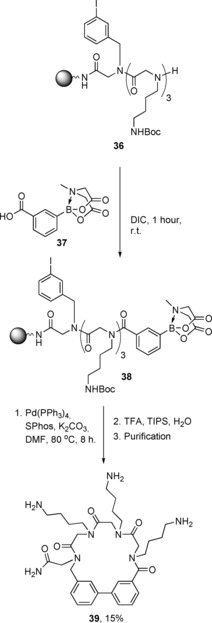
Synthesis of a biaryl‐containing macrocylic peptoid (**38**).


Recent work from within our own group has demonstrated peptoid cyclisation via an on‐resin Suzuki cross‐coupling reaction (Scheme 24). One of our aims in this work was the inclusion of a biaryl linkage within the cyclic peptoid, as such motifs are present in many therapeutics including antifungal, antitumour, anti‐inflammatory and antihypertensive agents.^60^ In order to achieve this, we had to include both an aromatic iodide and an aromatic boronic acid in the linear parent peptoid (**38**) The iodide was incorporated by using 3‐iodobenzylamine as a building block in sub‐monomer peptoid synthesis. The boronic acids, 3‐ or 4‐carboxyphenylboronic acid MIDA ester, were incorporated into the linear peptoids using solid‐phase peptide synthesis conditions (e.g. formation of **38** in Scheme 21). Cyclisation was then achieved by incubation of the resin‐bound linear parent peptoids (e.g. **38**) at 80 °C for 8 hours in the presence of tetrakis palladium, Buchwald′s ligand (SPhos) and potassium carbonate in DMF. Subsequent cleavage from the resin and HPLC purification yielded the biaryl‐containing cyclic peptoids (e.g. **39**, Scheme 21) in yields of 3–23 %.^61^ Hexameric cyclic biaryl peptoids (**39**) as well as the larger heptameric cyclic biaryl peptoids (e.g. **40**) were both successfully synthesised. Generally speaking, the cyclisation of the longer linear peptoids was less efficient. Biaryl cyclic peptoids with 3‐3 linkages (e.g. **39** and **40**) and 4‐4 linkages (e.g. **41**) were also successfully synthesised. It was found that for short linear peptoids the 4‐4 regio‐isomers cyclised more efficiently, whereas with the longer peptoid chains, regio‐isomerism appeared to make little difference.

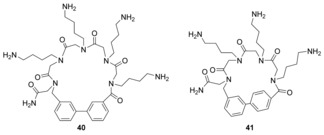


## 5 Summary and Outlook

Peptoids represent a promising class of peptidomimetics, retaining many of the advantages of peptides, such as biocompatibility and a high degree of chemical diversity, whilst being far more resistant to proteolytic degradation. However, the location of the peptoid side‐chains on the backbone amide nitrogen precludes any hydrogen bonding and as such peptoids display a high degree of conformational flexibility. Cyclisation is one approach that the peptoid community has adopted in an effort to access peptoids with more conformational rigidity. The latter is a highly desirable property in the development of therapeutic agents. Since the 2010 review of this area by Yoo and Kirshenbaum, several new methods of synthesising macrocyclic peptoids have been reported, bringing ready access to a new range of peptoid scaffolds. The synthesis of macrocyclic peptoids is an area that is likely to continue to grow. Molecules of this type offer up excellent opportunities for the design of new bioactive agents and they can be used as building blocks to access complex peptoid nano‐structures.

## Conflict of interest

The authors declare no conflict of interest.

## Biographical Information

*Alexandra M. Webster received her M.Chem. from the Chemistry Department at Durham University in 2013. She is currently pursuing a Ph.D. in Chemistry within the Cobb group at Durham University. Her Ph.D. research focuses on the development of new methodologies for the design and synthesis of biologically active peptoids. Specifically, she is looking into designing synthetic strategies to access new types of peptoid scaffolds*.

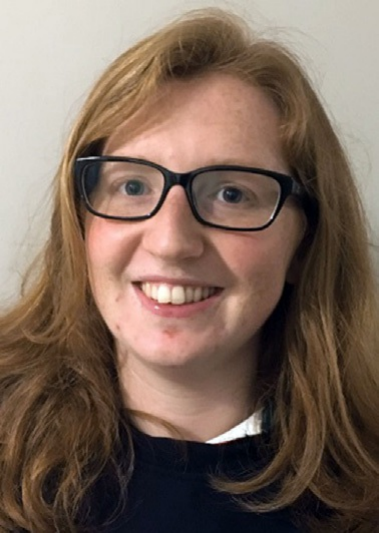

## Biographical Information

*Steven L. Cobb obtained his Masters in Chemistry at the University of St. Andrews. He then received his Ph.D. in Bioorganic Fluorine Chemistry at the University of St. Andrews, where he worked with Professor David O′Hagan on the biosynthesis of novel fluorinated natural products. After his Ph.D. he carried out postdoctoral studies on the development of new peptide based antibiotics with Professor John C. Vederas FRS at the University of Alberta, Canada. He returned to the UK (Durham University) in 2008 as a Ramsay Memorial Fellow and he is currently an Associate Professor of Chemical Biology and Director of the Centre for Global Infectious Diseases in the Chemistry Department. His research interests are focused on the development of new peptide and peptoid based therapeutics for the treatment of antimicrobial infections*.

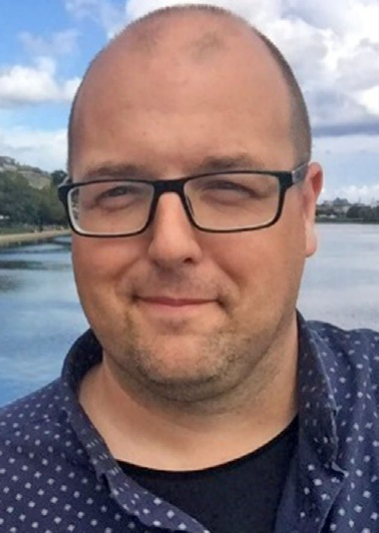

## Supporting information

As a service to our authors and readers, this journal provides supporting information supplied by the authors. Such materials are peer reviewed and may be re‐organized for online delivery, but are not copy‐edited or typeset. Technical support issues arising from supporting information (other than missing files) should be addressed to the authors.

Supplementary
