# Supplementary material for: Recent Advances in the Synthesis of Peptoid Macrocycles
Source: Chemistry. 2018 Feb 21;24(30):7560–73. doi: 10.1002/chem.201705340 (PMC6001806; doi:10.1002/chem.201705340)
Supplement: Supplementary file 1 — Supplementary [file CHEM-24-7560-s001.pdf]

Author Contributions

A.W. Conceptualisation: Equal; Investigation: Equal; Writing – original draft: Equal; Writing – review & editing: Supporting.
